# Integration of Provider, Pharmacy, and Patient-Reported Data to Improve Medication Adherence for Type 2 Diabetes: A Controlled Before-After Pilot Study

**DOI:** 10.2196/medinform.4739

**Published:** 2016-02-08

**Authors:** Brian E Dixon, Abdullah H Alzeer, Erin O'Kelly Phillips, David G Marrero

**Affiliations:** ^1^ Indiana University Richard M. Fairbanks School of Public Health Department of Epidemiology Indianapolis, IN United States; ^2^ Regenstrief Institute, Inc. Center for Biomedical Informatics Indianapolis, IN United States; ^3^ Center for Health Information and Communication Health Services Research and Development Service Department of Veterans Affairs, Veterans Health Administration Indianapolis, IN United States; ^4^ School of Informatics and Computing Department of BioHealth Informatics Indiana University Indianapolis, IN United States; ^5^ Diabetes Translational Research Center Indiana University School of Medicine Indianapolis, IN United States; ^6^ Department of Endocrinology Indiana University School of Medicine Indianapolis, IN United States

**Keywords:** medication adherence, barriers to medication use, diabetes mellitus, type 2, medical records systems, computerized, health records, personal, physician-patient relations, drug monitoring, patient-centered care

## Abstract

**Background:**

Patients with diabetes often have poor adherence to using medications as prescribed. The reasons why, however, are not well understood. Furthermore, most health care delivery processes do not routinely assess medication adherence or the factors that contribute to poor adherence.

**Objective:**

The objective of the study was to assess the feasibility of an integrated informatics approach to aggregating and displaying clinically relevant data with the potential to identify issues that may interfere with appropriate medication utilization and facilitate patient-provider communication during clinical encounters about strategies to improve medication use.

**Methods:**

We developed a clinical dashboard within an electronic health record (EHR) system that uses data from three sources: the medical record, pharmacy claims, and a patient portal. Next, we implemented the dashboard into three community health centers. Health care providers (n=15) and patients with diabetes (n=96) were enrolled in a before-after pilot to test the system’s impact on medication adherence and clinical outcomes. To measure adherence, we calculated the proportion of days covered using pharmacy claims. Demographic, laboratory, and visit data from the EHR were analyzed using pairwise t tests. Perceived barriers to adherence were self-reported by patients. Providers were surveyed about their use and perceptions of the clinical dashboard.

**Results:**

Adherence significantly and meaningfully improved (improvements ranged from 6%-20%) consistently across diabetes as well as cardiovascular drug classes. Clinical outcomes, including HbA1c, blood pressure, lipid control, and emergency department utilization remained unchanged. Only a quarter of patients (n=24) logged into the patient portal and completed psychosocial questionnaires about their barriers to taking medications.

**Conclusions:**

Integrated approaches using advanced EHR, clinical decision support, and patient-controlled technologies show promise for improving appropriate medication use and supporting better management of chronic conditions. Future research and development is necessary to design, implement, and integrate the myriad of EHR and clinical decision support systems as well as patient-focused information systems into routine care and patient processes that together support health and well-being.

## Introduction

### Type 2 Diabetes Mellitus

Type 2 diabetes mellitus (T2DM) is a major public health issue, affecting more than 350 million people worldwide and the fourth leading cause of death. Globally, the prevalence of T2DM continues to rise at nearly epidemic rates, driven by urbanization, growing increases in obesity, and aging of populations [1]. Findings from several studies investigating the quality of T2DM care reveal a discrepancy between system-level disease management strategies and outcomes [2-6]. In essence, even though there are improved treatment strategies, expected outcomes are not occurring at a commensurate level. Therefore, greater emphases on patient-level factors that may explain T2DM intervention outcomes are being explored.

### Patient Adherence to Medication

An example of a patient-level factor is adherence to complex medication regimens. Increasing evidence suggests that patients with T2DM often have poor adherence with prescribed medication therapies [7,8]. The reasons why individual patients do not take their medications as prescribed, however, are poorly understood. Existing research about medication adherence tends to investigate the issue as a class phenomenon, suggesting that patients as a group are universally impacted by a somewhat narrow range of factors such as side effects, cost, and forgetfulness [9].

Because adherence is treated as a class phenomenon, interventions tend to focus on singular modalities to change provider or patient behavior. This is especially true for informatics-related interventions. For example, in Vollmer et al [10], an interactive voice response system called patients who appeared to have gaps in refilling their asthma medication. Automated calls were made to patients or family members, but no assessment of individual barriers to adherence were measured or factored into the system. Similarly, recent systematic reviews of consumer-focused health information technologies conclude that prior studies tend to offer patients narrowly scoped functionalities out-of-the-box without regard to individual situations with limited success [11,12]. A recent review by Sapkota et al [13] found that while 22 of 52 (42%) interventions resulted in modest improvements in adherence, just 9 (17%) improved both adherence and glycemic control.

Despite existing efforts to improve adherence, patient-reported barriers to medication adherence (eg, lack of ability to pay, beliefs about the efficacy of medications in treating a condition, transportation to pharmacy, etc) and the extent to which those barriers contribute to poor T2DM outcomes are not currently assessed routinely in clinical practice [14]. Indeed, few have assessed the role of barriers perceived by patients to medications use and how perceived barriers may be addressed by intervention.

To address barriers to taking medications as prescribed facing individuals with T2DM, we developed a Web-based module for an electronic health record (EHR) system to electronically integrate the capture and presentation of information regarding T2DM patients’ disease management, medication adherence, and perceived barriers to adherence [15]. The system combines three elements: (1) objective data regarding medication possession ratios; (2) laboratory and point-of-care testing data that can indicate medication use; and (3) patient-reported data on perceived barriers to adherence. By routinely capturing patient-reported barriers and integrating such information with other electronic health data that is accessible during the clinical encounter, we seek to better potentiate patient-provider communication about medication use and thus inform T2DM therapy decision-making processes.

Following the development of the EHR module, we pilot tested the intervention in 3 primary care clinics located in an urban environment. By pilot testing the module, we sought to evaluate: (1) the extent to which patients would be willing to provide data to inform provider-patient conversations about medication use; (2) the impact of the system on medication use as prescribed by providers; and (3) the system implementation in a real-world clinical setting. We hypothesized that patients would be willing to share their perceived barriers to adherence as increasingly health systems are seeking patient input into clinical decision-making processes. We further hypothesized, given known challenges with adherence among patients with diabetes, that the intervention would improve adherence rates, which would improve other clinical indicators such as diabetes and cardiovascular risk factor control. Finally, we hypothesized that the dashboard would be used and positively perceived by providers.

### Study Aim

In this paper, we describe the results of the pilot testing. We further comment on the implications of the pilot for future research and development of integrated informatics solutions to support patient-centered care.

## Methods

### Setting

Eskenazi Health is one of the 5 largest safety net health systems in the United States. The health system contains a 315-bed hospital and 9 community health centers located across the metropolitan area of Indianapolis, the eleventh largest city in the United States. Each community health center provides adult primary care, pediatrics, obstetrics, gynecology, and mental health.

The lead author (BED) presented the design of the dashboard and study to health center leadership at a health system meeting, asking for volunteers. In this study, 5 of the 9 community health centers volunteered to participate. We purposely selected 3 of the 5 health centers to ensure geographical and socioeconomic diversity among the study patient population. Only 3 health centers were selected to best manage provider and patient enrollment.

### Study Participants

First, we recruited primary care providers with the authority to prescribe medications, which included medical doctors and nurse practitioners practicing in the 3 target clinics. Staff at ResNet, a practice-based research network recruitment engine used by Eskenazi as well as the Indiana Clinical and Translational Sciences Institute [16], approached eligible providers (n=29). Once providers gave their permission to participate, ResNet used the EHR to identify potential patients with diabetes for recruitment.

Provider participation was voluntary. Out of 29 eligible providers, 15 (52%) agreed to participate. Of the 14 eligible providers who did not participate, 8 never responded to our invitations to participate, 2 indicated they did not treat patients with T2DM, 1 indicated she was leaving the practice within 30 days, and 1 indicated she was not interested.

Potential patient participants were identified based on past medical history documented in the EHR. Queries of the EHR identified 2369 potential patients who were at least 18 years of age and possessed: (1) either an International Classification of Diseases, Version 9 or local diagnosis term indicating type 2 diabetes; (2) at least one active prescription for either biguanides, sulfonylureas, or thiazolidinediones (diabetic drug therapies); and (3) a history of primary care visits with an enrolled provider. ResNet staff then contacted eligible patients via phone to further screen them for enrollment. Screening questions asked patients to confirm they did have diabetes and were taking medication to manage their disease. In addition, phone screeners asked whether the patient possessed regular access to a computer with the Internet, as well as their ability to read English. After verification of inclusion criteria, phone screeners next described the study, and then asked for informed consent. Consenting patients were asked to provide either an email address or mobile phone number that could receive a text message to receive further instructions during the study. ResNet staff worked to contact, screen, and consent eligible patients until a sufficient number (n=96) was enrolled.

### System Description

**Figure 1:**
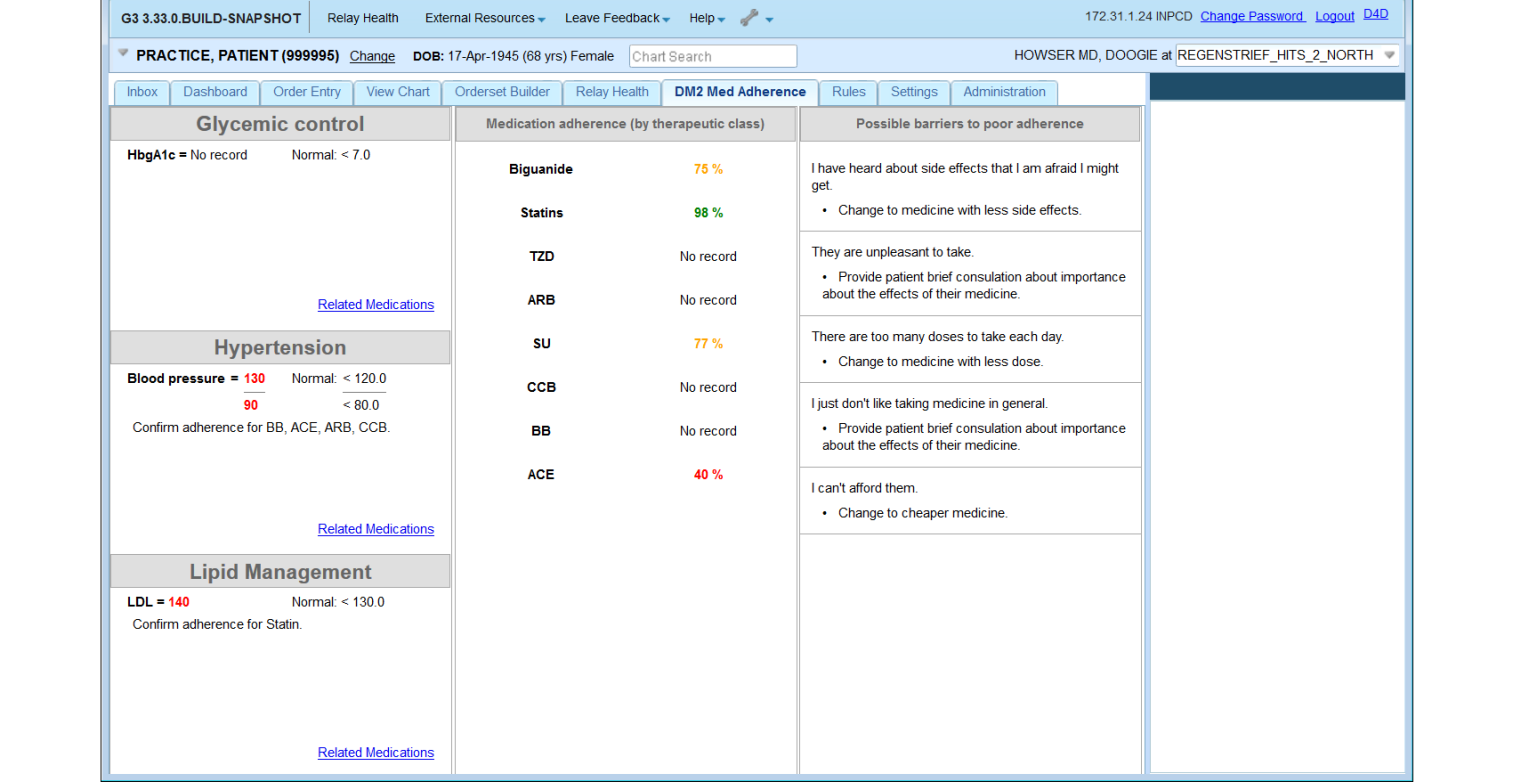
Screenshot of clinical dashboard designed to integrate medication adherence information from multiple sources into electronic health record (EHR) system.

The primary system of interest is a clinical dashboard (Figure 1 shows this) used by providers in the context of routine primary care. The dashboard is a Java-based module designed to plug into the Regenstrief CareWeb framework, an open-source EHR platform developed by the Regenstrief Institute’s Center for Biomedical Informatics. CareWeb is a Web-based version of the Regenstrief Medical Record System (RMRS), providing primary care clinicians in Eskenazi Health facilities access to patients’ medical records [17]. We have previously described the design and development of the dashboard [15].

When a clinician selects a patient in the EHR, the dashboard refreshes with content from multiple sources: recent physiological data from the EHR, pharmacy data providing objective medication adherence data from a medication module, and patient-reported barriers to medication adherence from a patient portal. Briefly, we review the dashboard components and data sources.

### Electronic Health Record Data

The left panel of the dashboard displays information extracted from the RMRS. There are three types of EHR data relevant to diabetic patient populations that are displayed: blood pressure, HbA1c, and cholesterol. Clinicians routinely measure and analyze HbA1c levels to determine how well diabetic patients keep their disease under control. High blood pressure and poor lipid management are also commonly corelated with type 2 diabetes, leading to polypharmacy regimens to treat multiple conditions.

### Medication Adherence Data

Adherence to T2DM and related medications is displayed in the middle panel of the dashboard. The information originates from the Medication (Med) Hub, an independent Web service within the Regenstrief technology infrastructure designed to gather and reconcile a patient’s current medication list [18]. Using available pharmacy data from the Med Hub, we calculate the proportion of days covered (PDC), a ratio representing whether the patient possessed a drug or a class of drugs (eg, all oral T2DM medications) during a defined measurement period. The PDC has been shown in numerous studies to accurately identify patients who fail to fill or refill their medications as directed by their physician or pharmacist [19], and it is the recommended measure for adherence by the Pharmacy Quality Alliance [20]. We use a dichotomized 6-month (180-day) PDC with a cut-off point of 80%, which we have found to provide the strongest and most reliable correlation with patient glycemic control [21].

### Patient-Reported Barriers to Adherence

**Figure 2:**
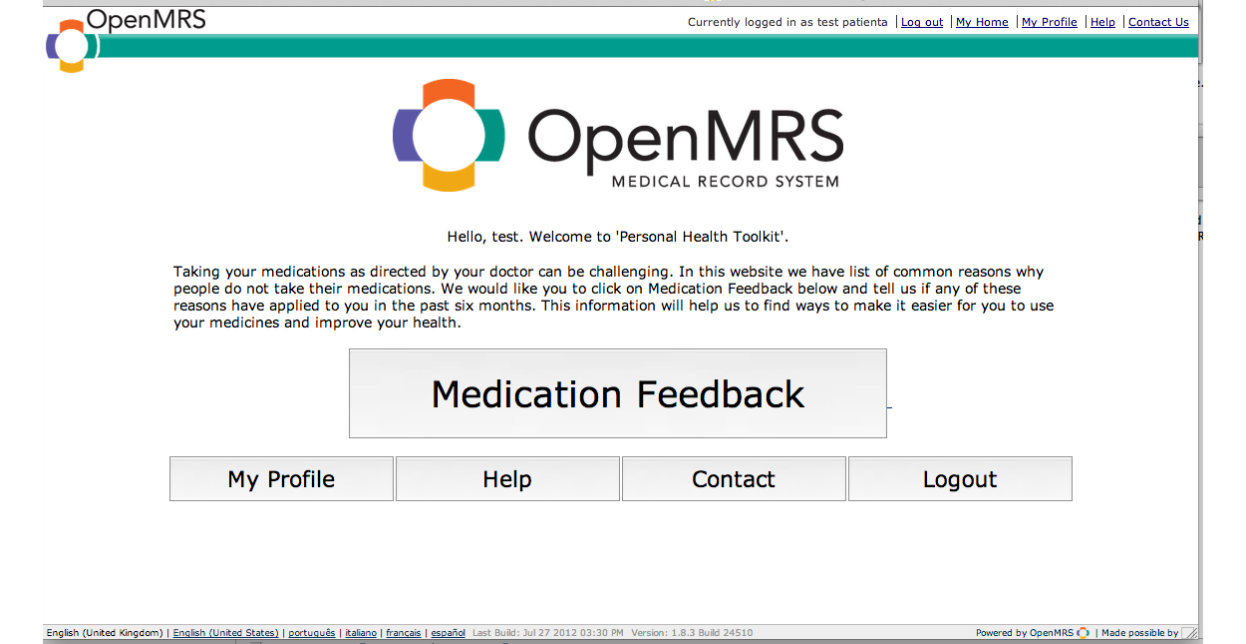
Screenshot of the patient portal displays the initial screen following log in which prompts the user to complete the questionnaire about medication usage and challenges.

The right panel of the dashboard displays patient-reported barriers to taking their medications as prescribed. Patients report their barriers to adherence using a Web-based portal (Figure 2 shows this welcome screen) developed using the Open Medical Record System (OpenMRS) platform [22], an open-source EHR that originated at Regenstrief, but is now implemented and supported by a worldwide collaborative involving individuals from numerous counties involved in EHR and m-Health initiatives [23]. OpenMRS includes a forms module that allows collection of standardized data from patients. Using the forms module, we implemented a 5-point Likert style, validated questionnaire developed by researchers at the Diabetes Translational Research Center affiliated with the Indiana University School of Medicine [24,25]. The questionnaire, as implemented in the portal, is included as Appendix 1 (see Multimedia Appendix 1).

The patient questionnaire uses 20 items to assess possible barriers to medication adherence. For example, valid responses as to why one may not take his or her prescribed medications include, “I can’t afford them” and “I just forget to take them”. There are 5 factors or subscales that can be identified from responses to the questionnaire and displayed to clinical users: poor access to medications; poor communication with providers; poor understanding of medications and difficulty taking them or difficulty in taking them; presence of side effects; and system-level barriers to use. Previous analysis suggests that persons with poor cardiovascular disease (CVD) risk factor control have more reported barriers that may inhibit medication adherence than do persons with good risk factor control [25].

### Information Flow

**Figure 3:**
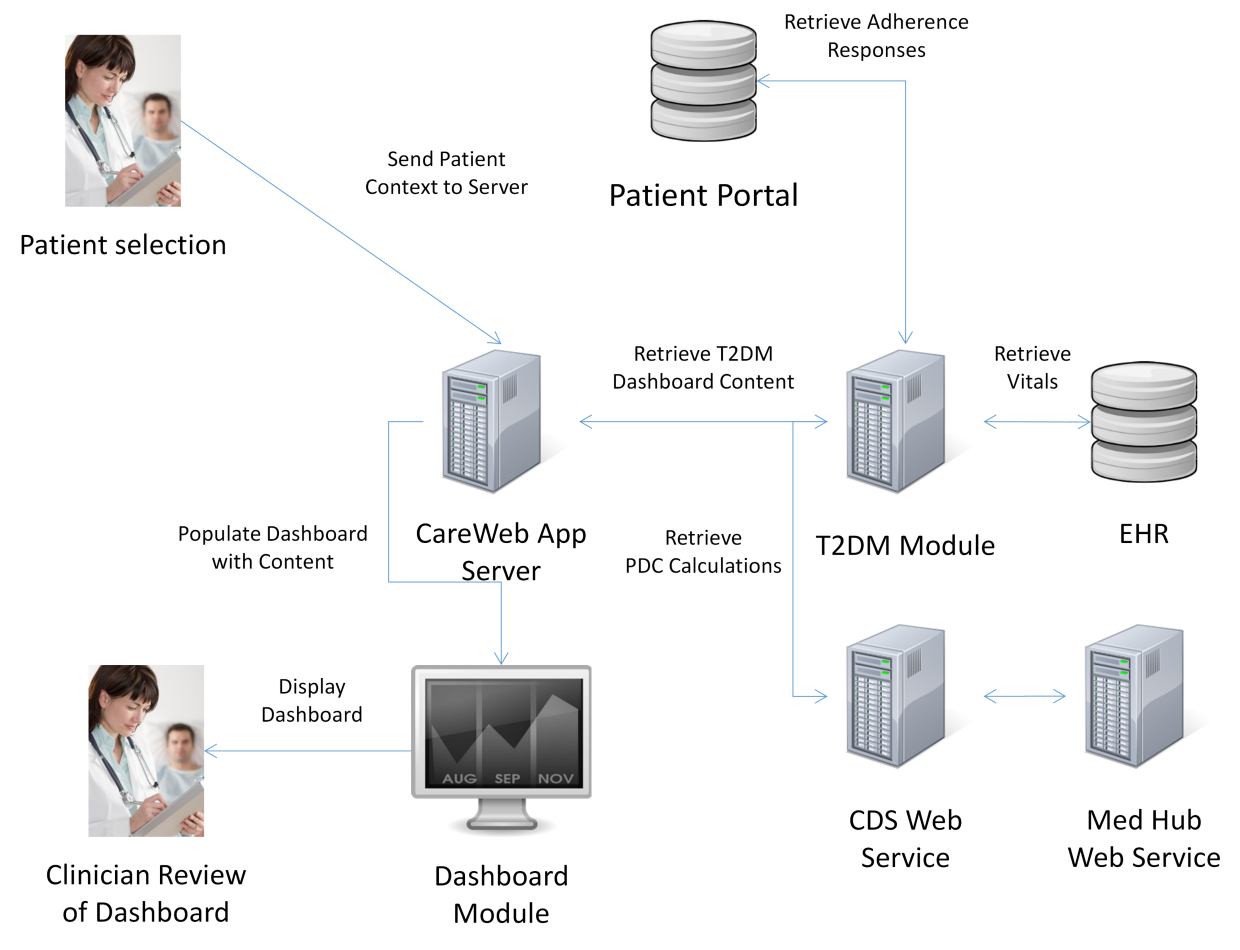
Information flow diagram depicting the architecture of the type 2 diabetes clinical information system module and its integration with existing electronic health record system, clinical decision support system, and patient portal components. T2DM = type 2 diabetes mellitus; EHR = electronic health record; CDS = clinical decision support; and Med = medication.

The information needed for the dashboard is queried in parallel with other CareWeb processes when a clinician opens the EHR for a patient (Figure 3 shows this). First, the CareWeb server notifies the T2DM module that a patient record has been selected. The module then, in parallel, requests data from the RMRS, Med Hub, and Web-based patient portal. The respective datasets are stored in the server’s cache until the clinician selects the “DM2 Med Adherence” tab within the CareWeb apps (Figure 1). Upon selection, the datasets are rendered into their respective columns for review by the clinician.

### Study Design

Following enrollment of providers and patients, a research assistant created patient portal user accounts for each of the enrolled patients. Account information was then emailed or texted to patients, and they were asked to complete a baseline questionnaire regarding perceived barriers to using medications as prescribed by their clinician. Patients who did not complete a baseline questionnaire were reminded via email or text message, based on patient preferences. Patients were further asked to complete a questionnaire every 2-3 months after baseline. Reminders were sent to unresponsive participants. After successful completion of each questionnaire, patients were provided a US $10 retail gift card.

Consenting providers were given access to the integrated clinical dashboard within their EHR. This meant the tab labelled “DM2 Med Adherence” in Figure 1 was enabled for their user profile in the EHR, allowing them to access it at any time. Providers were informed about the tab and offered a brief tutorial by a nurse informaticist who regularly visits the clinic to educate providers about changes to the EHR. In addition, noninterruptive reminders were displayed to enrolled providers when relevant data for a patient were available for review on the dashboard. Providers could open the dashboard by clicking on the reminder in addition to, or instead of, clicking on the “DM2 Med Adherence” tab shown in Figure 1.

To assess the effect and implementation of the module, we used a controlled before-after design. We evaluated: (1) the willingness of patients to provide data via the Web-based portal; (2) changes to patients’ medication adherence as well as clinical indicators; and (3) provider usage and perceptions of the module. To measure patient willingness to provide data, we captured data on patient enrollment in the study as well as completion rates of adherence barrier questionnaires during the pilot timeframe. To measure changes in medication adherence as well as clinical indicators, we compared patients’ adherence, diabetic control, lipid control, and health care utilization rates before and after the introduction of the dashboard. Baseline data were collected from participants’ medical records for the year prior to the introduction of the intervention. The same data were collected from participants’ medical records for the 9-month pilot study. To measure provider engagement with the EHR system, we captured data on whether and how often they accessed the dashboard. We further surveyed providers about their use and perceptions of the dashboard following the pilot. The study was reviewed and approved by the Institutional Review Board at Indiana University (Protocol No. 1109006851).

### Data Analysis

Descriptive statistics were calculated for participant characteristics as well as adherence, diabetic control, lipid control, and health care utilization variables. Mean values were calculated across all observations (eg, multiple HbA1c measurements) during the preintervention (one year before) and postintervention (9 months after) periods, and the means for each time period were compared using within-subject paired *t* tests. Adherence was measured using PDC calculated for each time period by patient for each drug class using the methods described in Nau [20] and Wang et al [26]. Patient-level PDC calculations were compared using within-subjects paired *t* tests. Patient-reported barrier data from questionnaires were summarized using descriptive statistics. Provider responses to questionnaires regarding their use and perception of the dashboard were summarized using counts and means; small numbers prevented the use of statistical analysis. All statistical tests were performed using SAS 9.4 (Carey, NC).

## Results

### Study Recruitment

Out of 2369 potential patients identified by the EHR system, we attempted to recruit 906 (38.24%) via telephone in an effort to reach our goal of 100 enrolled patients. A total of 203/906 (22.40%) patients completed screening, of which 131/203 (64.5%) were eligible. Those determined to be ineligible (n=72/131; 55.0%) reported that they did not have regular access to a computer or mobile device with access to the Internet or could not provide informed consent. All of the potentially eligible participants were patients with diabetes who were taking medications as forecasted by the EHR. Of the 131 patients eligible to participate, 108 (82.4%) consented to participate in the study and 96 (73.3%) completed enrollment procedures.

### Study Population Characteristics and Baseline Measures

Table 1 summarizes the characteristics of the final study population. African Americans were overrepresented given the population demographics of the Indianapolis metropolitan area from which they were selected. Most participants (n=84/96; 87%) were under 65, and half (n=50/96; 52%) possessed a baseline HbA1c above 8.0%, indicating they had difficulty controlling their diabetes. Participants were, on average, obese and possessed optimal low-density lipoprotein (LDL) cholesterol levels. On average, the participants visited their PCP (primary care provider) once every 3 months (mean >5 visits), and participants visited the emergency department (ED) once in the time period prior to the start of the intervention.

**Table 1:** Study population characteristics.

Characteristics		Count (%) or mean (median) n=96	SD
**Gender**			
	Male (%)	40 (42)	
	Female (%)	56 (58)	
**Race**			
	Caucasian (%)	47 (49)	
	African American (%)	41 (43)	
	Unknown (%)	8 (8)	
Age		53 (52)	11.00
HbA1c (%)		8.79 (8)	1.98
LDL (mg/dL)		95.6 (93)	34.18
Body mass index (kg/m^2^)		39.87 (37)	11.95
PCP visits		5.45 (5)	4.71
ED visits		1.02 (1)	1.35

### Postintervention Change to Adherence and Other Measures

Table 2 summarizes the change in physiologic, health care utilization, and PDC calculations observed 9-months after the introduction of the intervention. Participants were repeatedly invited to log in to the Web-based portal to complete the questionnaire about challenges they faced in taking their medications. Despite multiple prompts via email and short message service, only 24 participants completed at least one questionnaire during the study period. We therefore stratified the results based on whether the patient completed the questionnaire. However, we observed no significant differences in demographics (eg, gender, race) between patients who completed questionnaires and those who did not. We further observed little meaningful differences in clinical outcomes or health care utilization between those who did log in to the portal versus those who did not. However, medication adherence rates improved significantly and meaningfully across diabetes and cardiovascular drug classes.

**Table 2:** Patient engagement and outcomes measures.

Outcomes, means			Assessment completed^a^ (n=24)	No assessment completed^b^ (n=24)	Overall total (n=96)	Overall *P* ^c^
**Physiological**						
	**HbA1c**					
		Pre	8.71	8.82	8.79	.29
		Post	8.66	8.96	8.88	
	**LDL**					
		Pre	100.55	93.95	95.61	.06
		Post	100.71	109.29	107.27	
	**Body mass index**					
		Pre	40.41	39.71	39.87	.29
		Post	44.43	37.97	39.46	
**Utilization**						
	**PCP visits**					
		Pre	5.46	5.44	5.45	<.001
		Post	1.75	2.42	2.25	
	**ED visits**					
		Pre	1.00	1.03	1.02	.001
		Post	0.46	0.64	0.59	
**Diabetes drug classes, PDC %**						
	**PDC for biguanides (n=38)**					
		Pre	72	74	74	<.001
		Post	88	89	89	
	**PDC for thiazolidinediones (n=8)**					
		Pre	88	69	77	.004
		Post	96	91	93	
	**PDC for sulfonylureas (n=26)**					
		Pre	71	74	73	<.001
		Post	88	96	93	
**Cardiovascular drug classes, PDC %**						
	**PDC for ACE inhibitors (n=45)**					
		Pre	90	85	85	.04
		Post	83	85	91	
**PDC for angiotensin II receptor**						
	**Antagonists (ARB) (n=14)**					
		Pre	87	78	80	.02
		Post	96	92	93	
	**PDC for calcium channel blockers (n=18)**					
		Pre	92	85	87	.03
		Post	96	94	95	
	**PDC for beta blockers (n=30)**					
		Pre	86	73	75	<.001
		Post	94	90	91	
**PDC for 3-hydroxy-3-methyl-glutaryl CoA reductase**						
	**Inhibitors (statins) (n=13)**					
		Pre	80	80	80	.02
		Post	99	91	94	

^a^Patients who completed at least one Web-based assessment

^b^Patients who never logged in to complete an assessment

^c^Paired *t* test *P* value, overall

### Barriers to Adherence

The top 5 barriers, based on mean scores from the questionnaires completed by those patients who completed at least one questionnaire (n=24), are presented in Table 3. The mean scores for these items range from 5 (Rarely) to 6 (Sometimes). So while common, these items do not necessarily indicate the items impact adherence. The items range in nature from general to financial to possible side effects.

**Table 3:** Commonly reported perceived barriers to adherence by patients.

Questionnaire item	Mean score
I just don't like taking medicine in general.	5.69
I just forget to take them.	5.36
I can't afford them.	5.19
I ran out of medication before I could call or visit my doctor or nurse.	5.17
My medicines make me feel bad or have side effects I don't like.	5.15

### Feedback From Providers

Out of 29 eligible providers practicing at the 3 clinics prior to the implementation of the dashboard, 15 (52%) volunteered to participate. At the end of the 9-month study, 12 (80%) of these providers still practiced at the 3 participating sites. Out of the 12 providers still practicing, 6 (50%) provided feedback via a postintervention questionnaire.

All of the responding providers reported being aware of the dashboard when asked about it one month following the end of the pilot period. However, only 4 of the 6 (66%) reported using the dashboard at least once during the pilot. The 2 providers who indicated they did not use the dashboard responded negatively in general to questions about clinical information systems. For example, both these clinicians disagreed with statements that technology is easy to use in their workplace. They were further less than agreeable that technology could support care coordination or inform clinical decisions.

When asked about whether the dashboard was useful to patient-provider conversations about adherence and helpful to improving medication adherence or helpful to improving medication adherence, half of the providers responded negatively (eg, Disagree) and half were neutral. The providers who responded negatively to questions about the dashboard’s usefulness provided interesting open-ended comments. A provider reported that she was “not confident” regarding the quality of the information on the dashboard. Another respondent indicated he wasn’t sure if the dashboard contained data from outside pharmacies. A third provider recommended that patients “always bring their pill bottles” for independent verification of what they are taking, regardless of what data are displayed on the dashboard.

## Discussion

### Principal Results

A dashboard designed to inform clinicians and stimulate provider-patient conversations about medication adherence for patients with T2DM was introduced into the EHR system at 3 busy ambulatory clinics. Clinicians were prompted to review the dashboard for patients enrolled in a feasibility pilot, then work with their patients to address medication adherence issues illuminated by the dashboard. Following the introduction of the intervention, enrolled patients’ medication adherence improved significantly and meaningfully achieving its primary outcome. However, no significant changes to clinical outcomes or health care utilization were observed. This may be the result of patients being exposed to the barriers questionnaire; an exposure that may have stimulated their consideration of the importance of using medications as prescribed. In this regard, it is noteworthy that few patients were willing to complete Web-based questionnaires about their challenges to taking medications as prescribed using a Web-based portal from their home computer or mobile device. This may reflect concerns about divulging to their providers that they are not using medications as prescribed. The desire of patients to indicate positive medication use patterns even when in actuality they are not adhering to avoid social sanctioning has been noted in the medication adherence literature [5,8,13]. Provider usage was limited and perceptions were mixed. Therefore, while the intervention shows potential for addressing patterns of medication use, it will require further work, especially more effective implementation strategies, to more actively engage patients in contributing data and providers in using the data if broader impact beyond adherence is to be achieved.

Medication adherence improved following the intervention. For every drug class in which mean adherence was below 80% (the cut-off point considered “good” by the Physicians Quality Alliance) prior to the intervention, mean PDC was observed to be above 80% at the end of the study. Moreover, this trend was consistent for diabetes as well as CVD drug classes, for which adherence was also displayed on the dashboard given known comorbidity between diabetes and CVD. In fact, PDC improved not only in the overall cohort, but also across subgroups with one exception: PDC fell for patients taking ACE inhibitors who completed at least one Web-based questionnaire, although it did not dip below 80%. Thus, the dashboard appears to be effective at calling attention to adherence issues, even in cases where the dashboard lacked information on patients’ self-reported psychosocial challenges to taking their medications.

The combination of providing PDC alongside laboratory data may therefore be sufficient to stimulate patient-provider discussion about appropriate medication use. It is also possible that the requests for patients to provide data about barriers acted as a clinical reminder, which influenced patients to be mindful about medication use. Finally, it is plausible that patients, believing that their provider was going to see data about their medication utilization, elected to be more consistent in their medication use. Information on patient-provider conversations and motivations for better adherence were not captured in this study; these would be important dimensions to measure in a larger study involving mixed methods.

Although the primary objective was achieved, we did not observe any meaningful changes to patients’ health status. Diabetic control and BMI remained unchanged, and health care utilization remained constant. With respect to diabetic control, in theory, this should improve in parallel with medication adherence. Where patients with diabetes use their medications as directed by clinicians, HbA1c levels are expected to fall below 8.0%. A reason we may not have observed a change is the length of the pilot. It can take up to 3 months for HbA1c values to change following a change in medication usage, and only a few patients had more than 2 measured HbA1c values in the EHR system by the end of the 9-month pilot. With respect to BMI, we did not anticipate a change based on the intervention and included drug classes. When considering indicators associated with CVD (eg, LDL), values postintervention trended in the wrong direction, although the change was not quite statistically significant. This was surprising given that adherence for CVD drug classes also increased. Finally, with respect to health care utilization, although the *P* values were significant, clinically the changes are insignificant. Patients continued to see their PCP approximately every 3 months, and ED visits averaged one per year when you adjust the values for the shortened postintervention observation period.

### Limitations

Results of the pilot should be interpreted with caution given the small size of the cohort and limited timeframe of the study. Informatics interventions can take a while to be adopted and routinely used by clinicians. In our study, only half of the clinicians practicing in each health center agreed to participate in the study. Not surprisingly, many clinicians we approached who are experiencing serious time constraints, were hesitant about adopting yet another tool into their routine workflow. Other clinicians were near retirement, and some did not want to be bothered with participation in any research study. Given mixed participation and limited use, the intervention may not be directly responsible for changes in adherence especially since our model did not control for other factors. A future trial of such an intervention in the larger health system would need to control for patient as well as provider and clinic factors to be more confident in stating the effect of the informatics intervention.

In addition, the project struggled to engage patients in logging into the portal where they could complete the psychosocial questionnaire about barriers to taking their medications. Even with a financial incentive, just one-quarter of the cohort completed at least one questionnaire during the 9-month pilot. Many enrolled patients struggled due to computer or Internet access issues. For example, one participant repeatedly stated she was waiting for her daughter to come over to help her. Other patients’ email addresses bounced less than 1-2 weeks after they provided them during the enrollment process. Text messaging and phone calls were helpful in reaching some of these patients, but access challenges remained for a significant number of study subjects. Furthermore, the patient portal was not routinely used by our clinical partner, Eskenazi Health. The portal vendor for the health system refused to work with the study team, stating their platform was designed only for secure messaging and they did not have an interest in expanding their service offerings to enable patient reported data to be integrated into the EHR. Therefore, the clinicians were unfamiliar with the portal, which may have contributed to lackluster participation by patients beyond their Internet access challenges.

### Comparison With Prior Work

Prior studies to improve adherence and glycemic control or glycemic control tend to focus on singular modalities to change provider or patient behavior with limited success. For example, in Vollmer et al [10], an interactive voice response system called patients who appeared to have gaps in refilling their asthma medication. The system was statistically significant in changing adherence, but the mean change (2%) was not clinically meaningful (eg, impact on health outcomes as well as quality of life). Similarly, a systematic review of patient portals by Ammenwerth et al [12] identified just one study that demonstrated an effect on diabetes care delivery. While that one portal was found to be associated with a change in medication regimen, it had no impact on clinical outcomes as measured by HbA1c and blood pressure [27]. A recent systematic review by Sapkota et al [13] of interventions targeting patients with type 2 diabetes found that just 9 of 52 (17%) studies found an improvement to both adherence and glycemic control. A broader meta-analysis of consumer-focused health information technology trials by Or and Tao [11] similarly found that none of the identified studies showed an impact on clinical or psychosocial outcomes; and the impact of patient-centered technologies had mixed effects on consumer behavior. While our intervention did have both statistically significant and clinically meaningful impact on adherence, our impact on more core measures of quality were similarly disappointing.

### Future Directions

Given the context of prior studies, our results suggest a change to how we design, implement, and integrate technologies into care delivery systems and consumers’ lives. Our dashboard received positive feedback from some of the clinicians before and during the study. Yet to access the dashboard, clinicians needed to click on yet another tab within the EHR system or be prompted to view the dashboard when data were available for a given patient. While alerts can be useful to guide provider behavior, they can also be viewed as a nuisance leading to providers ignoring or overriding them [28,29]. This may be why some providers reported not using the dashboard during the pilot. Therefore, as we develop dashboards or other EHR widgets, we must find ways to more seamlessly integrate them into clinical processes when contextually appropriate. Other projects at Regenstrief have explored contextually sensitive alerts [30-32], concluding they are promising. Furthermore, we need to explore other clinical team roles (eg, medical assistant, registered nurse, clinical pharmacist), beyond the physician, for whom an adherence dashboard might make more sense given the workflow in primary care as well as other settings.

In addition to information and workflows, we can refine our approach to synthesis as well as visualization of information in clinical dashboards. For example, in this study, patient reported barriers were often missing, yet medication adherence improved. Therefore, it may be sufficient to provide a view of adherence using just 2, objective data sources (pharmacy claims, vital signs). This would assume, however, that providers trust the data and dashboard. In this feasibility pilot, 2 of the providers were wary of the data in the dashboard. Better awareness and experience with integrated data views might solve this issue; or better strategies to educate providers on the sources and quality of data in EHR systems might be considered.

Moreover, when patient barriers were missing, a full third of the screen was blank. Other visualizations of adherence data in combination with vitals sign trends and patient-reported data or patient-reported data could be explored to design EHR widgets that might reduce the potential for white space or maximize screen real estate. A larger trial could explore, for example, variants to the information visualization tested here to find optimal representations of multiple data streams.

To be effective, “routine clinical care” must also include environments where patients exist (eg, home, work, bus stop). Integrated information solutions, therefore, must incorporate technologies that can be integrated into daily routines of people. Our efforts were hampered by system barriers that remain a challenge for many people, especially those who are elderly or of low socioeconomic status [33]. Thus, a digital divide still exists, even if it may be shrinking in populations burdened by multiple comorbid diseases requiring complex drug regimens. Therefore, future studies should consider approaches that can reach broad populations instead of developing on an isolated platform that cannot be integrated into the existing health information infrastructure. A pilot study involving text messages to homeless veterans that reduced appointment no-show rates [34] and the multiple studies involving text4baby [35] demonstrate that even disadvantaged populations can benefit from health informatics interventions delivered in a modality that is broadly accessible to patients. Instead of the Web-based browser-based portal we used, we could have explored mobile platforms that could be accessed via mobile phones. Yet one-dimensional “smart” apps are similarly unlikely to be sufficient. The patient-focused aspect of our pilot was largely one-sided, designed to collect information from patients instead of engage them in learning about their disease and strategies for self-management. Management of diabetes involves not only medication adherence, but also changes to diet, exercise, and comorbid conditions such as hypertension. Therefore, technologies that engage patients will likely be those that can address multiple concerns in an integrated fashion (eg, one-stop shop) rather than require multiple apps or interfaces. In addition, it will become critical that future apps include appropriate education that emphasizes the importance of patient-provider communication to enable shared decision making that will achieve optimal therapeutic outcomes.

### Conclusions

To fully realize the potential of health information technologies to support patient-centered care delivery, while impacting population health outcomes, information and technical systems need to be integrated. Our vision of technical interfaces among EHR, CDS, and patient information systems, which are integrated into clinical and personal ecosystems, is necessary to create environments in which shared decision making can be informed by evidence, an individual’s health data, and knowledge of social determinants. The results from our early pilot of an integrated approach are promising. Yet, they suggest additional research and development to better design, implement, and integrate the myriad of EHR and CDS systems, as well as patient devices, into routine care and patient processes that together support health and well-being.

## Appendix

Multimedia Appendix 1 Questionnaire given to patients regarding perceived barriers to taking their medications as prescribed.

## Abbreviations

BMI: body mass index
CVD: cardiovascular disease
ED: emergency department
EHR: electronic health record
LDL: low-density lipoprotein
MED: medication
OpenMRS: Open Medical Record System
PCP: primary care provider
PDC: proportion of days covered
RMRS: Regenstrief Medical Record System
T2DM: type 2 diabetes mellitus
