# Supplementary material for: Integration of Provider, Pharmacy, and Patient-Reported Data to Improve Medication Adherence for Type 2 Diabetes: A Controlled Before-After Pilot Study
Source: JMIR Med Inform. 2016 Feb 8;4(1):e4. doi: 10.2196/medinform.4739 (PMC4763113; doi:10.2196/medinform.4739)
Supplement: Multimedia Appendix 1 [file medinform_v4i1e4_app1.pdf]

OpenMRS

Currently logged in as test patienta | Log out | My Home | My Profile | Help | Contact Us

Discard changes | Print

test patienta | Medication Barriers Form (Unsaved Form)

Save Changes

1.	I just forget to take them.	Never	Rarely	Sometimes	Often	Very Often*
2.	I forgot to fill my prescription in time.	Never	Rarely	Sometimes	Often	Very Often*
3.	I don't know what does to take.	Never	Rarely	Sometimes	Often	Very Often*
4.	I'm not sure exactly what each medicine is for.	Never	Rarely	Sometimes	Often	Very Often*
5.	There are too many doses to take each day.	Never	Rarely	Sometimes	Often	Very Often*
6.	It's too hard to keep track of what I am supposed to take when.	Never	Rarely	Sometimes	Often	Very Often*
7.	They are unpleasant to take.	Never	Rarely	Sometimes	Often	Very Often*
8.	I can't afford them.	Never	Rarely	Sometimes	Often	Very Often*
9.	My medicines make me feel bad or have side effects I don't like.	Never	Rarely	Sometimes	Often	Very Often*
10.	I have heard about side effects that I am afraid I might get.	Never	Rarely	Sometimes	Often	Very Often*
11.	Getting to the pharmcy to pick them up is difficult.	Never	Rarely	Sometimes	Often	Very Often*
12.	The pharmacy could not fill my prescription.	Never	Rarely	Sometimes	Often	Very Often*
13.	My doctor or nurse forgot to write a new prescription.	Never	Rarely	Sometimes	Often	Very Often*
14.	I ran out of medication before I could call or visit my doctor or nurse.	Never	Rarely	Sometimes	Often	Very Often*
15.	I don't have enough time to talk with my doctor or nurse about problems I am having with my medicines.	Never	Rarely	Sometimes	Often	Very Often*
16.	I sometimes forget to ask my doctor or nurse about prblems that I am having with my medicines.	Never	Rarely	Sometimes	Often	Very Often*
17.	I don't feel my medicines are helping me.	Never	Rarely	Sometimes	Often	Very Often*
18.	I just don't like taking medicine in general.	Never	Rarely	Sometimes	Often	Very Often*
19.	Taking medicines makes my health worse.	Never	Rarely	Sometimes	Often	Very Often*
20.	I sometimes find it hard to ask my doctor or nurse questions about my medications.	Never	Rarely	Sometimes	Often	Very Often*

Enter Form
